# Diabetes-Specific Questionnaires Validated in Brazilian Portuguese: A Systematic Review

**DOI:** 10.20945/2359-3997000000216

**Published:** 2020-03-18

**Authors:** Leonardo Grabinski Bottino, Mariana Migliavacca Madalosso, Sheila Piccoli Garcia, Beatriz D. Schaan, Gabriela Heiden Teló

**Affiliations:** 1 Faculdade de Medicina Universidade Federal do Rio Grande do Sul Porto Alegre RS Brasil Faculdade de Medicina da Universidade Federal do Rio Grande do Sul (UFRGS), Porto Alegre, RS, Brasil; 2 Hospital de Clínicas de Porto Alegre Porto Alegre RS Brasil Hospital de Clínicas de Porto Alegre (HCPA), Porto Alegre, RS, Brasil; 3 Faculdade de Nutrição Universidade Federal do Rio Grande do Sul Porto Alegre RS Brasil Faculdade de Nutrição da Universidade Federal do Rio Grande do Sul (UFRGS), Porto Alegre, RS, Brasil; 4 Programa de Pós-Graduação em Endocrinologia Universidade Federal do Rio Grande do Sul Porto Alegre RS Brasil Programa de Pós-Graduação em Endocrinologia, Universidade Federal do Rio Grande do Sul (UFRGS), Porto Alegre, RS, Brasil; 5 Divisão de Endocrinologia Hospital de Clínicas de Porto Alegre Universidade Federal do Rio Grande do Sul Porto Alegre RS Brasil Divisão de Endocrinologia, Hospital de Clínicas de Porto Alegre, Universidade Federal do Rio Grande do Sul (UFRGS), Porto Alegre, RS, Brasil; 6 Instituto Nacional de Ciência e Tecnologia de Avaliação em Tecnologias em Saúde Conselho Nacional de Desenvolvimento Científico e Tecnológico Porto Alegre RS Brasil Instituto Nacional de Ciência e Tecnologia de Avaliação em Tecnologias em Saúde (IATS) – Conselho Nacional de Desenvolvimento Científico e Tecnológico (CNPq), Porto Alegre, RS, Brasil

**Keywords:** Brazil, diabetes mellitus, surveys, questionnaires

## Abstract

Two researchers conducted independent searches on five different electronic databases: PubMed/MEDLINE, Embase, SciELO, LiLACS and Web of Science. Studies were selected that covered cross-cultural adaptation methodology and validation in Brazil with type 1 and type 2 diabetes patients of any age. After reading the full-text articles, data related to psychometric characteristics were extracted from each study selected. Reliability was assessed with Cronbach’s α (Cα). The initial searches identified 2,211 studies. After exclusions, 26 were included, covering a total of 31 questionnaires. Questionnaires were grouped into 11 domains based on their main focus of interest: adherence (n = 8), quality of life (n = 7), diabetes knowledge (n = 3), hypoglycemia (n = 3), self-efficacy (n = 3), satisfaction with pharmaceutical services (n = 1), emotional stress (n = 2), hope (n = 1), attitude towards diabetes (n = 1), perception of disease severity (n=1), and risk of developing diabetes (n = 1). This study identified and reviewed all of the diabetes-specific questionnaires that have been validated for Brazilian Portuguese, which should facilitate selection of the most appropriate instrument for each domain of interest in future research and clinical settings.

## INTRODUCTION

Recent data show that the global prevalence of diabetes increased from 108 million in 1980 to 425 million in 2017 (1,2). It is estimated that by 2045, 629 million people will be affected with diabetes worldwide (2). Brazil is the country with the fourth highest number of diabetes cases in the world, with a prevalence of 12.5 million people with diabetes in 2017 (2), which has been increasing significantly over the last 35 years (3).

The special and rigorous care that is necessary for good glycemic control affects several spheres of patients’ lives, such as eating, physical activity, quality of life, and mental health, among others. This characteristic of the disease requires frequent monitoring by health professionals, preferably with a multidisciplinary approach (4). Additionally, diabetes is a disease with great potential to disable, due to its complications (5), which means that frequent evaluations of every aspect of the disease are needed (4). Questionnaires, scales, and other instruments have proved to be important tools for evaluating many chronic diseases, including diabetes, in both clinical practice and research settings (6). Questionnaires are great tools for collecting information about people’s behavior, knowledge and attitudes, by administering standardized questions (7).

Questionnaires can be classified as general questionnaires, which evaluate health variables and allow comparison between patients and healthy persons, or specific questionnaires, which evaluate characteristics related to a specific disease (8). Diabetes-specific instruments are very well accepted by patients and enable evaluation of specific aspects of the disease (9). These tools have been identified as one of the best ways to evaluate certain characteristics of diabetes, such as diabetes burden and mental health (10).

The number of questionnaires cross-culturally adapted and validated for the Brazilian culture is still insufficient for the high diabetes demands in Brazil (10). There are no studies comparing all the available instruments, which could facilitate selection of the most appropriate instrument for each domain of interest in future research and in the clinical setting. Therefore, the present study aims to identify and compare all diabetes-specific questionnaires validated in Brazilian Portuguese.

## SUBJECTS AND METHODS

This systematic review protocol was registered on the *International Prospective Register of Systematic Reviews* (PROSPERO) under registration number CRD42017073407. This protocol is fully available on the National Institute of Health Research – Health Technology Assessment Database (NIHR HTA) website. This systematic review follows the recommendations of the Preferred Reporting Items for Systematic Reviews and Meta-Analyses (PRISMA) (11).

### Information sources

The literature search was conducted independently by two reviewers on five electronic databases (Medical Literature Analysis and Retrieval System Online/PubMed, EMBASE, Web of Science, Scientific Electronic Library Online [SciELO], and Latin American and Caribbean Center on Health Sciences Information [LILACS]) in order to identify diabetes-related questionnaires, scales, and other instruments that have been translated, adapted, and validated for the Brazilian culture. Other mechanisms such as Google Scholar were also used in order to reduce publication bias and include unpublished data or publications not indexed in the databases listed above. Manual searches were also conducted in the references of the articles selected and in the annals of major Brazilian and international conferences related to diabetes held during the last three years. Where studies were identified, but the full text was unavailable, the authors were contacted in order to request the full article. The most recent search was performed on October 3^rd^, 2017.

### Search strategy

Descriptors “Diabetes”, “Questionnaires”, and “Brazil” and related terms and keywords were used in the search strategy, in English and Portuguese. The search strategy run on PubMed is described in Table 1.

**Table 1:** Literature Search Strategy Used for PubMed Database

**#1** Search: ((((((“Diabetes Mellitus”[Mesh]) OR “Diabetes Mellitus, Type 2”[Mesh]) OR “Diabetes Mellitus, Type 1”[Mesh]) OR “Diabetes Mellitus, Lipoatrophic”[Mesh] OR Diabetes OR Diabetic*)) OR LADA[Title/Abstract]) OR MODY[Title/Abstract]
**#2** Search:((((((((((((((((((“Surveys and Questionnaires”[Mesh]) OR ( “Validation Studies as Topic”[Mesh] OR “Validation Studies” [Publication Type])) OR “Translations”[Mesh])) OR adaptation[Title/Abstract]) OR reliability[Title/Abstract])) OR translation[Title/Abstract]) OR scale[Title/Abstract]) OR transculturalization[Title/Abstract]) OR questionnaire[Title/Abstract]) OR validation[Title/Abstract]) OR cross-cultural[Title/Abstract]) OR validity[Title/Abstract]) OR version[Title/Abstract]) OR validation studies[Publication Type]) OR instrument[Title/Abstract]) OR score[Title/Abstract]) OR cultural[Title/Abstract]
**#3** Search: ((Brazil* OR Brasil OR “Minas Gerais” [Title/Abstract] OR “São Paulo” [Title/Abstract] OR “Espirito Santo” [Title/Abstract] OR “Rio de Janeiro” [Title/Abstract] OR Bahia [Title/Abstract] OR Pará [Title/Abstract] OR “Mato Grosso” [Title/Abstract] OR “Mato Grosso do Sul” [Title/Abstract] OR Goiás [Title/Abstract] OR “Rio Grande do Sul” [Title/Abstract] OR Ceará [Title/Abstract] OR Pernambuco [Title/Abstract] OR “Santa Catarina” [Title/Abstract] OR Amazonas [Title/Abstract] OR Maranhão [Title/Abstract] OR Tocantins [Title/Abstract] OR Piauí [Title/Abstract] OR Rondônia [Title/Abstract] OR Roraima [Title/Abstract] OR Paraná [Title/Abstract] OR Acre [Title/Abstract] OR Amapá [Title/Abstract] OR Paraíba [Title/Abstract] OR “Rio Grande do Norte” [Title/Abstract] OR Alagoas [Title/Abstract] OR Sergipe [Title/Abstract] OR “Distrito Federal” [Title/Abstract] OR Portuguese[Title/Abstract] OR Portugues[Title/Abstract] OR Português[Title/Abstract]”))
**#4** Search: #1 AND #2 AND #3

### Eligibility criteria

Studies were selected based on the following criteria: translation to Brazilian Portuguese, cross-cultural adaptation, or validation of questionnaires, scales, and other instruments related to diabetes. Only patient-reported outcome measures were included. A decision was taken to exclude articles in which the instrument validated required additional information, such as the results of laboratory procedures, imaging examinations, or careful professional evaluation, thereby ruling out inclusion of risk scores. No date or language restrictions were applied.

### Study selection

Duplicates were identified and excluded. Two researchers (L.G.B. and M.M.M.) independently evaluated titles and abstracts against the inclusion and exclusion criteria. Publications for which the information needed could not be extracted from the title or abstract were selected for full article reading. All studies selected in the first evaluation were referred for analysis of the full text. Disagreements were resolved by arriving at a consensus in discussions or by a third researcher (G.H.T). Agreement between the two researchers was analyzed using the kappa coefficient and a value > 0.80 was defined as almost perfect agreement (12). EndNote version X7 software (Thomson Reuters, New York, NY) was used to organize the studies.

### Data extraction

Two researchers (L.G.B. and M.M.M.) separately extracted the following data from each study: title in Portuguese and English, publication date, authors, instrument name in English and Portuguese, population used for validation, target age, cross-cultural adaptation process, reliability data, type of validation, instrument application time, mode of administration (self-report or administered by an interviewer), instrument accessibility, number of items and domains, scores and cut-off points. Disagreements were resolved by discussion and, if unresolved on this basis, a third researcher (G.H.T.) was consulted. When two studies validated the same questionnaire in a similar population but with different sample sizes, only the most accurate was included. Data were inputted to a pretested Microsoft Office Excel^TM^ spreadsheet.

### Risk of bias in studies

The quality of studies was evaluated using the Cochrane tool (13). Certain adaptations were made in order to use it for validation studies, because it was originally created for evaluating randomized clinical trials. The bias risk assessment took into account presence of important methodological elements of the cross-cultural adaptation/validation process: validation additional to the cross-cultural adaptation process (14), reliability data, test-retest evaluation, pre-test evaluation, and sample size calculation or estimation.

Reliability was measured with Cronbach’s α. In general, the minimum acceptable value for the reliability of a questionnaire is 0.70; below this value, the internal consistency of the scale is considered low. In contrast, the expected maximum value is 0.90; above this value, one might consider redundancy or duplication, which means that several items would be measuring exactly the same element of a construct; therefore, redundant items should have been eliminated. Usually, Cronbach’s α values between 0.80 and 0.90 are preferred (8,15). We used the value 0.7 to consider a questionnaire acceptably reliable.

## RESULTS

**Figure 1:**
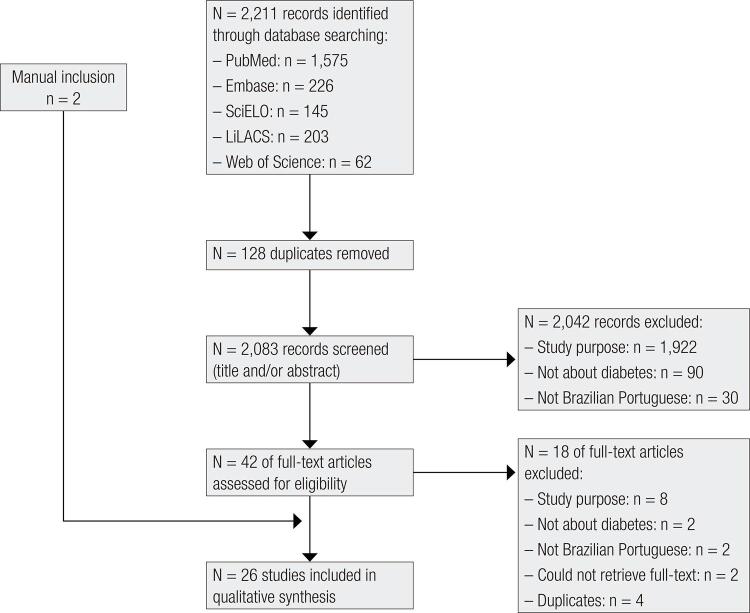
Flow diagram: identification and selection of studies included in the systematic review.

The initial searches identified 2,211 articles; 128 were duplicates and were excluded; 2,083 articles were analyzed by title and abstract, and 43 were selected for full-text reading. Two papers were found only in abstract form. Since their validation studies were not found, the authors were contacted and requested to provide them. Both (16,17) had used unvalidated instruments. In both cases, the instruments were excluded because it was impossible to analyze the studies. Twenty-four studies were included initially, and another two studies were found by manual searching, totalling a final sample of 26 studies included (Figure 1). Although not fully published, three important questionnaires related to hypoglycemia were included since they presented sufficient information and psychometric data in the abstract (18).

The total number of instruments adapted/validated was 31, because some articles used more than one questionnaire. Some of these validations were conducted for the same questionnaire, but in different populations, such as type 1 and type 2 diabetes [the Self-Care Inventory-revised questionnaire (19,20) and the Diabetes Quality of Life Measure (21,22)] or different age ranges (19,23), totaling 28 different instruments.

With regard to the populations used to validate the instruments, one study stated that the questionnaire had been validated for patients with both types of diabetes (type 1 and type 2 diabetes), but the sample included only one patient with type 2 diabetes (24). In this case, we included this study in the systematic review as exclusively validated for patients with type 1 diabetes. An analysis of concordance between the researchers was conducted, generating a Kappa coefficient of 0.848 (P < 0.001).

In order to facilitate understanding and enable comparisons between the instruments selected, the researchers grouped the studies into domains based on the main subject addressed. This resulted in 11 large domains representing the primary focus of each questionnaire: treatment adherence (n = 8, Cronbach’s α = 0.71-0.79 for type 1 diabetes mellitus; Cronbach’s α = 0.63-0.84 for type 2 diabetes mellitus); quality of life (n = 7, Cronbach’s α = 0.70-0.94 for type 1 diabetes mellitus; Cronbach’s α = 0.70-0.94 for type 2 diabetes mellitus); diabetes knowledge (n = 3, Cronbach’s α = 0.75-0.91 for type 1 diabetes mellitus; Cronbach’s α = 0.81 for health professionals); hypoglycemia (n = 3, Cronbach’s α = 0.73-0.84 for type 1 diabetes mellitus); self-efficacy (n = 3, Cronbach’s α unavailable for type 1 diabetes mellitus; Cronbach’s α = 0.63-0.78 for type 2 diabetes mellitus); satisfaction with pharmaceutical services (n = 1, Cronbach’s α unavailable for type 2 diabetes mellitus); emotional stress (n = 2, Cronbach’s α = 0.93 for type 1 diabetes mellitus; Cronbach’s α unavailable for type 2 diabetes mellitus); hope (n = 1, Cronbach’s α = 0.83 for type 2 diabetes mellitus); attitude towards diabetes (n = 1, Cronbach’s α = 0.91 for type 2 diabetes mellitus); perception of disease severity (n= 1, Cronbach’s α = 0.66 for both type and type 2 diabetes mellitus); and risk of developing diabetes (n = 1, Cronbach’s α unavailable for type 2 diabetes mellitus). Half of the studies reporting overall reliability had instruments with Cronbach’s α between 0.7 and 0.9. More details on the studies included in this systematic review and the instruments validated are presented in table 2.

**Table 2:** Characteristics of the instruments according to the approach, population studied, items and domains, and psychometric data

Area of interest	Author year	Instruments (original)	Instruments (Portuguese)	Population	Items domains	Application time	Cronbach’s α	Accessibility
**Adherence to treatment**	Stacciarini, T. 2014 (25)	Appraisal of Self Care Agency Scale-Revised (ASAS-R)	NA	T2DM	15 items	5 min	0.74	NA
	Telo, G. H. 2014 (19)	Diabetes Self-Management Profile (DSMP)	NA	T1DM	24 items 5 domains	20-30 min	0.76	NA
	Passone, C.G.B. 2017 (23)	Diabetes Self-Management Profile (DSMP)	NA	T1DM	25 items 5 domains	20 min	0.79	NA
	Boas, L. C. 2014 (26)	NA	Medida de Adesão aos Tratamentos – Antidiabéticos orais (MAT ADOs)	T2DM	7 items	61 min	0.84	NA
	Boas, L. C. 2014 (26)	NA	Medida de Adesão aos Tratamentos – insulina (MAT Insulina)	T2DM	7 items	61 min	0.68	NA
	Telo, G. H. 2014 (19)	Self-Care Inventory-revised (SCI-R)	NA	T1DM	14 items	8-10 min	0.71	NA
	Telo, G.H. 2017 (20)	Self-Care Inventory-revised (SCI-R)	NA	T2DM	15 items	8-10 min	0.63	NA
	Michels, M. J. 2010 (27)	Summary of Diabetes Self-Care Activities (SDSCA)	Questionário de Atividades de Autocuidado com o Diabetes (QAD)	T2DM	15 items (+3 ad)	7-15 min	NA	Available online
**Attitude**	Torres, H.C. 2005 (28)	Attitudes Questionnaires (ATT-19)	NA	T2DM	19 items 6 domains	20-30 min	0.91	NA
**Evaluation of hypoglycemia**	Giaretta, L.S. 2016 (18)	Reduced Awareness of Hypoglycemia (Clarke score)	NA	T1DM	NA	NA	0.73	NA
	Giaretta, L.S. 2016 (18)	Edinburgh Hypoglycemia Symptom Scale	NA	T1DM	NA	NA	0,84	NA
	Giaretta, L.S. 2016 (18)	Hypoglycemia Awareness Status (Gold Method)	NA	T1DM	NA	NA	NA	NA
**Emotional Stress**	Curcio, R. 2012 (29)	Diabetes Distress Scale (DDS)	NA	T2DM	17 items 4 domains	NA	NA	NA
	Silveira, M.S. V.M. 2017 (30)	Type 1 Diabetes Distress Scale (T^1^DDS)	NA	T1DM	28 items	NA	0.93	NA
**Hope**	Sartore, A.C. 2008 (31)	Herth Hope Index (HHI)	Escala de Esperança de Herth (EEH)	T2DM	12 items	NA	0.834	Available online
**Knowledge**	Torres, H.C. 2005 (28)	Diabetes Knowledge Scale (DKN-A)	NA	T2DM	15 items 5 domains	20 - 30 min	0.91	NA
	Coutinho, L.L. 2015 (32)	The Diabetes: Basic Knowledge Test (DBKT)	NA	health professional	41 items 2 domains	NA	0.81	NA
	Souza, J. G. 2016 (33)	Spoken Knowledge in Low Literacy Patients with Diabetes(SKILLD)	NA	T2DM	10 items	5-10 min	0.75	NA
**Quality of life**	Gross, C. C. 2007 (34)	PAID (Problem Areas in Diabetes)	Versão Brasileira do PAID (B-PAID)	T2DM	20 items 4 domains	5-10 min	0.93	Available online
	Correr, C. J. 2008 (21)	Diabetes Quality of Life Measure (DQOL)	DQOL - Brasil	T2DM	44 items 4 domains	10-15 min	0.92	Available online
	Brasil, F. 2014 (22)	Diabetes Quality of Life Measure (DQOL)	DQOL- Brasil	T1DM	44 items 4 domains	20-40 min	0.94	Available online
	Brasil, F. 2015 (35)	Diabetes Quality of Life Measure (DQOL)	DQOL-Brasil-8	T1DM and T2DM	8 items	20-40 min	0.702	NA
	Novato, T. S. 2008 (36)	Diabetes Quality of Life for Youths (DQOLY)	Instrumento de Qualidade de Vida para Jovens com Diabetes (IQVJD)	T1DM	51 items 3 domains	NA	0.9333	NA
	Queiroz, F. A. 2009 (37)	Diabetes – 39 (D-39)	NA	T2DM	39 items 5 domains	NA	0.917	NA
	Xavier, A. T. 2011 (38)	Neuropathy and Foot Ulcer - Specific Quality of Life (NeuroQol)	NA	T1DM and T2DM	35 items 6 domains	NA	0.94	NA
**Perception of disease severity**	Lopes, I. de M. 2017 (24)	Perception of Severity of Chronic Illness (PSCI)	NA	T1DM and T2DM	14 items 6 domains	3.49 min	0.66	NA
**Risk of diabetes**	Cruz, P.A. 2010 (39)	NA	Questionário de Risco para Diabetes Mellitus (QRDM)	T2DM	7 items	11 min	NA	Available online
**Satisfaction**	Correr, C. J. 2009 (40)	Pharmacy Services Questionnaire (PSQ)	Questionário de Satisfação com os Serviços da Farmácia (QSSF)	T2DM	20 items 2 domains	NA	NA	NA
**Self-efficacy**	Pace, A. E. 2017 (41)	Diabetes Management Self-efficacy Scale for Patients (DMSES)	Escala de Autoeficácia no Controle do Diabetes para Pacientes	T2DM	20 items 4 domains	40 min	0.78	NA
	Chaves, F. F. 2017 (42)	Diabetes Empowerment Scale - Short Form (DES - SF)	Escala de Autoeficácia em Diabetes – Versão Curta (EAD-VC)	T2DM	8 items 8 domains	5-10 min	0.634	Available online
	Gastal, D. A. 2007 (43)	Insulin Management Diabetes Self-efficacy (IMDSES)	Escala de autoeficácia no manejo da insulina	T1DM	20 items 3 domains	NA	NA	Available online

NA: not available, T1DM: type 1 diabetes mellitus, T2DM: type 2 diabetes mellitus.

**Figure 2:**
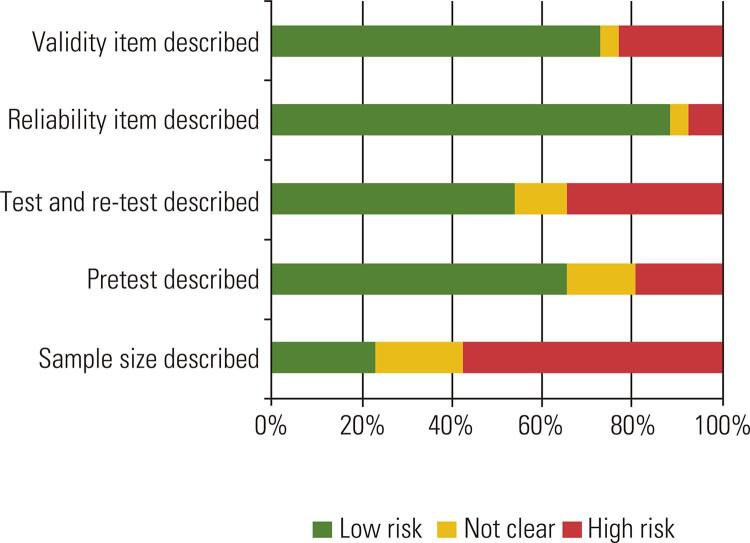
Within Group Bias Risk Assessment.

Analyses of the risk of bias within studies found that most studies presented a low risk of bias in the methodological steps and psychometric data evaluated (Figure 2 and Table 3). However, most of the studies lacked a methodological basis for determining sample size. Sample size was calculated in only five of the 26 studies (19,38,41-43), which may have modified the psychometric data and constitutes an increased risk of bias. Also, during the data extraction phase, there were some difficulties in extracting certain information due to lack of clarity in the descriptions of the methodological processes.

**Table 3:** Methodological steps and psychometric data bias risk assessment

Author Year	Sample size described	Pretest described	Test and re-test described	Reliability item described	Validity item described
Torres, H.C. 2005 (28)	-	**+**	**+**	**+**	**+**
Gastal, D.A. 2007 (43)	**+**	**+**	**+**	**?**	**+**
Gross, C.C.2007 (34)	-	-	-	**+**	**+**
Correr, C.J. 2008 (21)	-	-	-	**+**	**+**
Novato T.S. 2008 (36)	-	**+**	**+**	**+**	**+**
Sartore, A.C. 2008 (31)	-	**+**	**+**	**+**	**+**
Queiroz, F.A. 2009 (37)	-	**+**	**?**	**+**	**+**
Correr, C.J. 2009 (40)	**?**	-	-	**+**	**+**
Cruz, P.A. 2010 (39)	-	**+**	**+**	-	-
Michels, M.J. 2010 (27)	-	**+**	**+**	**+**	**+**
Xavier, A.T. 2011 (38)	**+**	**+**	**+**	**+**	**+**
Curcio, R. 2012 (29)	-	**+**	-	-	-
Boas, L.C. 2014 (26)	-	**?**	**?**	**+**	**+**
Brasil, F. 2014 (22)	-	**?**	**?**	**+**	**+**
Stacciarini, T. 2014 (25)	-	**+**	**+**	**+**	**+**
Telo, G. H. 2014 (19)	**+**	**+**	**+**	**+**	**+**
Brasil, F. 2015 (35)	**?**	**+**	**+**	**+**	**+**
Coutinho, L.L. 2015 (32)	**?**	-	-	**+**	-
Telo, G.H. 2017 (20)	**?**	**+**	**+**	**+**	**?**
Souza, J. G. 2016 (33)	-	-	-	**+**	-
Giaretta, L.S. 2016 (18)	**?**	**?**	-	**+**	-
Chaves, F.F. 2017 (42)	**+**	**+**	**+**	**+**	**+**
Lopes, I. de M. 2017 (24)	-	**+**	-	**+**	**+**
Pace, A.E. 2017 (41)	**+**	**+**	-	**+**	**+**
Passone, C.G.B. 2017 (23)	-	**+**	**+**	**+**	-
Silveira, M.S.V.M. 2017 (30)	-	**+**	**+**	**+**	**+**

**+** = low risk of bias, - = high risk of bias, **?** = bias unclear

## DISCUSSION

Due to the increasing number of diabetes cases in Brazil, it has been necessary to develop specific instruments to assist health professionals to follow-up these patients. The present study analyzed diabetes-specific questionnaires available and validated in Brazilian Portuguese with the purpose of facilitating the process of choosing the best surveys to use in clinical settings and research. A total of 26 studies were found that validated one or more instruments, totalling 31 different questionnaires validated in different populations (children/adolescents or adults with type 1 diabetes or type 2 diabetes).

There is a discussion in the literature about using disease-specific questionnaires rather than general questionnaires (8,44). It is agreed that general instruments are useful for the comparison between individuals with and without disease. However, concern has been expressed that a generic health measure would not be sensitive enough to capture all aspects of patient experience, and that a diabetes-specific measure would be needed to evaluate the overall impact of the disease and interventions. More specific instruments have the advantage of better assessing the unique aspects of a particular disease as well as its impact on patients’ lives (15), such as questions asking patients with diabetes about the burden of using insulin in public.

Adherence to treatment was the area with the greatest number of questionnaires. This is defined as behavior of accepting and following recommendations (such as using medication correctly, sticking to the correct diet, practicing physical activity, etc.) provided by a health care professional (45). The importance of measuring adherence is to assess whether and to what extent recommendations are followed by the patient. Of the questionnaires covering this subject, the Measure of Adherence to Oral Antidiabetic Treatments (MAT ADO) has the best reliability. However, this questionnaire only evaluates adherence to oral medications and takes a long time to administer (61 minutes) (26). For a global evaluation of adherence, including items such as physical exercise, blood glucose testing, insulin use, and diet, the Diabetes Self-Management Profile seems to be better for assessing adherence in children and adults with type 1 diabetes (19,23). The Self-Care Inventory-revised (SCI-R) is a widely-used treatment adherence questionnaire and may be the best instrument to assess a population consisting of both type 1 and type 2 diabetes patients (19,20).

Regarding quality of life assessment, a considerable number of questionnaires assess global quality of life or quality of life in relation to a specific complication, such as diabetic neuropathy (38). Health-related quality of life is a broad and subjective concept involving physical, psychological, and social factors, as well as the individual’s perception of their role in society (46). It is important, since treatment should not aim merely to control a particular element of a disease (such as control of glycated hemoglobin and avoiding complications related to diabetes), but should also target aspects related to improvement in quality of life and well-being (47). The Diabetes Quality of Life (DQOL) questionnaire is a comprehensive questionnaire that assesses satisfaction with treatment care and the impact of diabetes on various areas of life such as social life, family, physical activity, sexual life, etc. There are 4 versions validated for Brazilian Portuguese, including both type 1 and type 2 diabetes patients, as well as a specific evaluation for young people, all with adequate reliability and validation items (21,22,35,36).

Another essential subject to be measured in patients with diabetes is their knowledge about their disease, since it is directly associated with adherence to treatment (48). The Diabetes Knowledge Scale (DKN-A) questionnaire was one of the first diabetes-specific questionnaires to be adapted for the Brazilian population and remains a tool of great utility and excellent reliability (28).

Hypoglycemia plays an important role in management of patients with diabetes, especially those with type 1 diabetes undergoing intensive treatment (49). Questionnaires to measure hypoglycemia objectively have been available in other countries for decades; in Brazil, three tools have recently been validated and will probably be of great utility for our population (18).

The remaining concepts measured are also of great importance in the research and clinical follow-up of patients with diabetes: attitude towards diabetes, emotional distress, hope, perception of disease severity. However, some common barriers faced by patients with diabetes, such as beliefs, relationship with health care professionals, lifestyle changes (50), physical activity (51), and risk of driving (52) have not been evaluated in Brazilian diabetic populations and still require cross-cultural adaptation and validation in order to be ready for using.

This study has some limitations. First, it is limited to the evaluation of specific questionnaires in the population with diabetes, which may limit comparisons to other populations. Also, when analyzing the time taken to administer the instruments, it appears likely that some studies reported the total duration of the interviews conducted for validation of the instrument (including informed consent procedures, sociodemographic data collection, etc.), rather than only the time needed to administer the instrument itself. Because of this, we believe that the administration times for some of the instruments shown in table 1 may be overestimated. Additionally, some questionnaires were not fully validated, but translated and culturally adapted using only a pre-test evaluation, which left them with no available reliability information. Nevertheless, we believe this study extends the literature by identifying and bringing together all of the questionnaires that have been validated in Brazilian Portuguese.

In conclusion, this systematic review identified and analyzed all the diabetes-specific questionnaires that have been validated in Brazilian Portuguese in order to assist health professionals in follow-up and treatment of patients with diabetes, in both clinical and research settings.
